# PTK6 Promotes Cancer Migration and Invasion in Pancreatic Cancer Cells Dependent on ERK Signaling

**DOI:** 10.1371/journal.pone.0096060

**Published:** 2014-05-01

**Authors:** Hiroaki Ono, Marc D. Basson, Hiromichi Ito

**Affiliations:** Department of Surgery, Michigan State University, College of Human Medicine, East Lansing, Michigan, United States of America; Institute of Medical Science, University of Tokyo, Japan

## Abstract

Protein Tyrosine Kinase 6 (PTK6) is a non-receptor type tyrosine kinase that may be involved in some cancers. However, the biological role and expression status of PTK6 in pancreatic cancer is unknown. Therefore in this study, we evaluated the functional role of PTK6 on pancreatic cancer invasion. Five pancreatic cancer cell lines expressed PTK6 at varying levels. PTK6 expression was also observed in human pancreatic adenocarcinomas. PTK6 suppression by siRNA significantly reduced both cellular migration and invasion (0.59/0.49 fold for BxPC3, 0.61/0.62 for Panc1, 0.42/0.39 for MIAPaCa2, respectively, *p<0.05* for each). In contrast, forced overexpression of PTK6 by transfection of a PTK6 expression vector in Panc1 and MIAPaCa2 cells increased cellular migration and invasion (1.57/1.67 fold for Panc1, 1.44/1.57 for MIAPaCa2, respectively, *p<0.05*). Silencing PTK6 reduced ERK1/2 activation, but not AKT or STAT3 activation, while PTK6 overexpression increased ERK1/2 activation. U0126, a specific inhibitor of ERK1/2, completely abolished the effect of PTK6 overexpression on cellular migration and invasion. These results suggest that PTK6 regulates cellular migration and invasion in pancreatic cancer via ERK signaling. PTK6 may be a novel therapeutic target for pancreatic cancer.

## Introduction

Pancreatic cancer is the fourth leading cause of cancer mortality in the United States. [1] The outcome of patients with pancreatic cancer has been dismal with a 5% 5-year-survival rate. [2] The lethality of pancreatic cancer is due to its aggressive biological behaviors including a great potential for invasion and metastasis, and resistance to currently available anti-cancer agents. The molecular mechanisms responsible for these characteristics is largely unknown, and must be understood to improve the treatment outcomes of patients with pancreatic cancer.

Protein tyrosine kinase 6 (PTK6), or breast tumor related kinase (BRK) is a non-receptor type tyrosine kinase. It is related to the c-Src kinase family, possessing SH2 and SH3 domains. [3] PTK6 promotes cell differentiation in normal epithelial cells and is barely expressed in mature epithelial cells in gastrointestinal tract, breast or skin. [3]–[5] Although aberrant overexpression of PTK6 has been identified in several epithelial cancers, including cancers of the breast, lung, melanoma, prostate, colon, and ovary, the functions of PTK6 in cancer biology have not been fully characterized. [3], [6]–[12].

In this study, we evaluated the role of PTK6 on pancreatic cancer cell invasion and explored the downstream signals that might mediate such an effect. Our findings suggest that PTK6 regulates invasiveness by activating ERK and raises the possibility that PTK6 may be an important new molecular target to improve the efficacy of therapy for pancreatic cancer.

## Materials and Methods

### Materials

Culture media, fetal bovine serum (FBS) and penicillin/streptomycin (P/S) were obtained from Sigma-Aldrich (St. Louis, MO). Anti-PTK6 antibody was obtained from Santa Cruz Biochemistry (Santa Cruz, CA), anti-p44/42 ERK1/2, anti-phospho-p44/42 ERK1/2 (Thr202/Tyr204), anti-p38 MAPK, anti-phospho-p38 MAPK (Thr180/Tyr182), anti-STAT3, and anti-phospho-STAT3 (Tyr705) antibodies were obtained from Cell Signaling Technology (Danvers, MA), and anti-β-actin antibody was obtained from Sigma-Aldrich. The selective ERK1/2 inhibitor U0126 was obtained from Sigma-Aldrich.

### Human Tissues

Pancreatic cancer tissue slides were obtained from Department of Pathology at Sparrow Hospital, Lansing MI. The use of archived specimens for this study was approved by Michigan State University (MSU) and Sparrow Hospital Institutional Review Boards (IRBs). Our IRB committee waived the need for consent. Nine patients who underwent pancreatic resection for pancreatic ductal adenocarcinoma from 2002 though 2012 were selected and were included in this study. The archived formalin-fixed, paraffin-embedded specimens were sectioned on a rotary microtome at 4 µm’s. Enzyme induced epitope retrieval was performed by 0.03% protease E for 10 minutes at 37°C. Anti-PTK6 antibody was diluted in 1/100 with Normal Antibody Diluent (NAD)(Scytek – Logan, UT) and incubated for 1 hour at room temperature. Antigen-antibody reactions were visualized with the avidin-biotin-peroxidase complex system (R.T.U. Vectastain Elite ABC Reagent; Vector Laboratories, Burlingame, CA, USA). The slides were reviewed and PTK6 expression was graded according to cytoplasmic staining intensity as follows; negative, no staining or weak intensity staining in less than 5% of cells; weak to moderate positive, weak to moderate intensitiy; strong positive, strong intensity.

### Cell Cultures

Human pancreatic cancer cell lines of BxPC3, Capan1, Hs766T, Panc1, and MIA PaCa2 were obtained from the American Type Culture Collection. BxPC3 was maintained in RPMI 1640 medium supplemented with 10% FBS and 1% P/S in a humidified (37°C, 5% CO_2_) chamber. The other cell lines were maintained in DMEM medium containing 10% FBS and 1% P/S.

### Western Blot Analysis

Cells were lysed in cell lysis buffer (Cell Signaling Technology) with 1 mM Phenylmethanesulfonyl Fluoride (Cell Signaling Technology). The protein concentration of each cell lysate was estimated by BCA assay (Pierce Chemical). Cell lysates containing total 20 µg of protein were applied to 10% SDS-PAGE and transferred to polyvinylidene difluoride (PVDF) membranes (Invitrogen, Grand Island, NY). After being blocked with Tris buffered saline with 0.2% Tween-20 (TBST) containing 5% BSA or skim milk for 4 hours at room temperature, the membranes were incubated with antibody in appropriate dilution at 4°C. Horseradish peroxidase–conjugated donkey anti–rabbit IgG or sheep anti-mouse IgG (GE Healthcare, Piscataway, NJ) were used as secondary antibodies and incubated with the membranes at a 1/3000 dilution for 1 hour. β-actin was used as a loading control marker for normalization of each lane.

### Gene Silencing by Small Interfering RNA

Loss-of-function analysis was performed using siRNAs targeting PTK6 (s11487, Ambion, Grand Island, NY: sense 5′-CAUCCAUGGUUAAGUCAUAtt-3′, antisense 5′-UAUGACUUAACCAUGGAUGaa-3′) and negative control (Silencer Select Negative Control #2 siRNA, Ambion). Another pair of siRNAs targeting PTK6 and negative control were used (PTK6 and negative control, Invitrogen, St Louis, MO: Stealth RNAi-PTK6HSS183907 sense 5′-CAGGCUGUGCGGCACUACAAGAUCU-3′, antisense- 5′-AGAUCUUGUAGUGCCGCACAGCCUG-3′ and Stealth RNAi Negative Control Medium GC Duplex, respectively). Each siRNA (10 nmol/l) was transfected into pancreatic cancer cells using Lipofectamine RNA iMAX (Invitrogen) according to the manufacturer’s instructions. The knockdown of a target gene was confirmed at 96 hours by western blotting.

### Overexpression of PTK6 by Stable Transfection

Human PTK6 cDNA (cloneID: 5746034) was purchased from Open Biosystems (Pittsburg, PA) and amplified by PCR using primers (5′-CCCAAGCTTATGGTGTCCCGGGACCAGGC, and 3′-CGGGATCCTCAGGTCGGGTTCTCGTAGC). The PCR product was inserted into expression vector pcDNA3.1-myc/His B (Invitrogen) according to the manufacturer’s protocol. The established PTK6 expression vector was subjected to DNA sequencing analysis to confirm the correct insert of full lengths of PTK6 cDNA. PTK6 expression vector or corresponding empty vector were transfected into pancreatic cancer cells using Lipofectamine 2000 (Invitrogen) according to the manufacturer’s instructions. After 72 hours transfection, cells were incubated in culture medium containing appropriate concentrations of G418. By culture in selection medium containg G418 over 2 weeks, stable transfectant cells were selected out. The expression of PTK6 protein was subsequently confirmed by western blotting.

### Transwell Migration and Invasion Assay

Transwell migration and invasion assays were carried out in 24-well modified Boyden chambers precoated with (invasion) or without (migration) Matrigel (transwell-chamber, BD Biosciences, San Jose, CA,). Pancreatic cancer cells (BxPC3 1×10^5^ cells, Panc1, 5×10^4^ cells, and MIAPaCa2, 7.5×10^4^ cells per well, respectively) in serum-free medium were seeded onto the trans-membrane in the upper chamber with 10% FBS in the lower chamber as a chemoattractant. After a 24 hour incubation, the cells that had migrated through the membrane were fixed and stained with the Diff-Quik Stain Set (Siemens, Newark, DE). They were then counted under magnification in 5 randomly selected high power fields. Each assay was performed in triplicate.

### Statistical Analysis

Comparison of the continuous values was performed using Student’s t-test and 2-sided *p*-values<0.05 were considered significant.

## Results

### PTK6 Expression Status in Pancreatic Cancer

**Figure 1:**
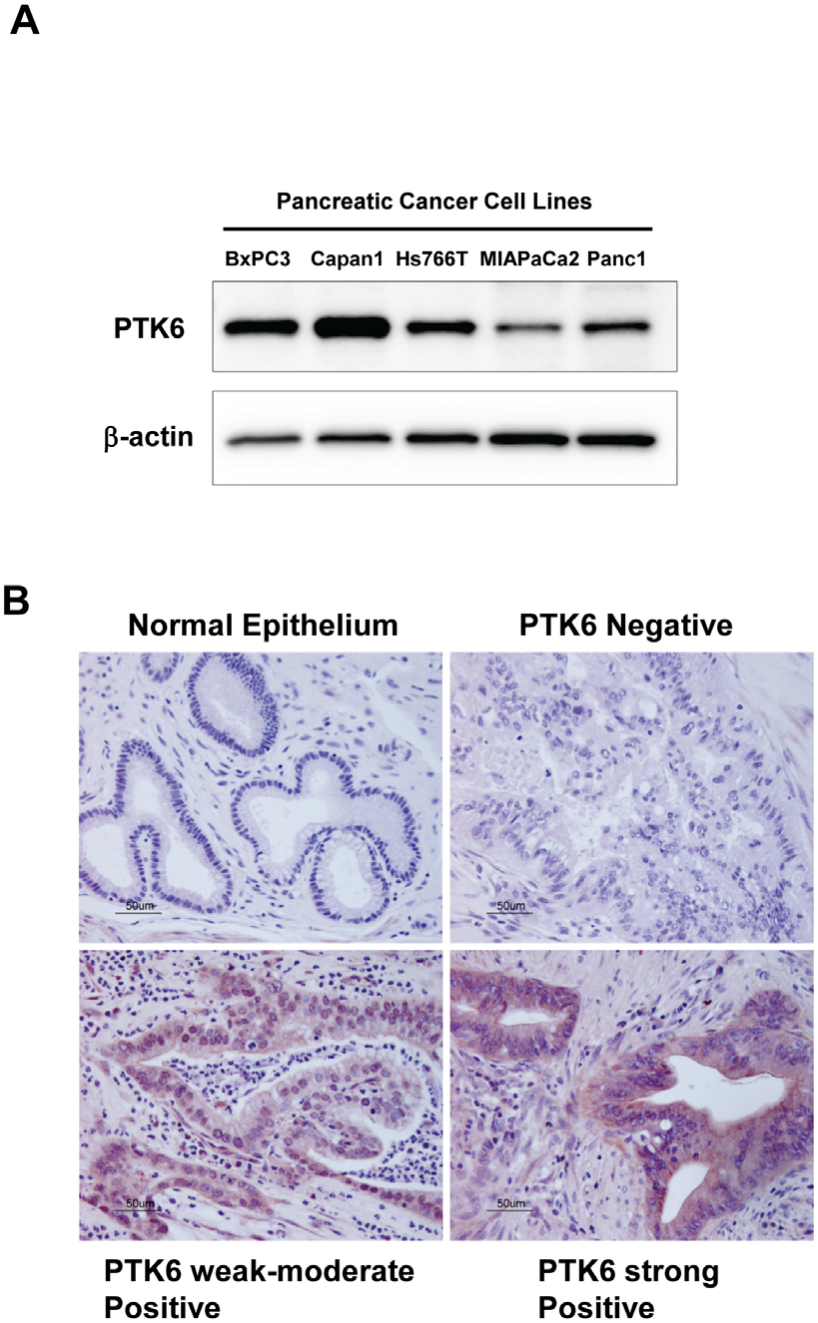
PTK6 is expressed in pancreatic cancer cell lines and human pancreatic adenocarcinoma tissues at various levels. ***A*,** Endogenous expression of PTK6 in human pancreatic cancer cell lines. PTK6 expression was confirmed in 5 pancreatic cancer cell lines, BXPC3, Capan1, Hs766T, MIAPaCa2, and Panc1 at various levels by Western-blotting. ***B*** **,** Immunohistochemical staning for PTK6 in adjacent normal pancreas and pancreatic cancer tissue. While there was no detectable PTK expression in adjacent normal pancreatic ductal epithelium (upper left), PTK6 was expressed at various levels (from negative through strongly positive) in pancreatic cancers.

We compared PTK6 levels in five established human pancreatic cancer cell lines (BXPC3, Capan1, Hs766T, MIAPaCa2, and Panc1) and pancreatic cancer tissues taken directly from 9 patients who underwent surgical resection. Western blots demonstrated that the 5 pancreatic cell lines expressed PTK6 at various levels; BXPC3 and Capan1 expressed PTK6 robustly, while MIAPaCa2 expressed a much lower level of PTK6 (Figure 1A). Immunostaining for PTK6 in pancreatic cancer tissues from 9 patients demonstrated that PTK6 was highly expressed in 4 patients (44%), mildly to moderately expressed in 2 patients (22%), and not expressed at all in 3 patients (33%). In the adjacent normal pancreatic duct epithelium, immunoreactive PTK6 was not detectable in any of the specimens we examined (Figure 1B).

### PTK6 Expression Affects Cell Migration and Invasion of Pancreatic Cancer Cell Lines

**Figure 2:**
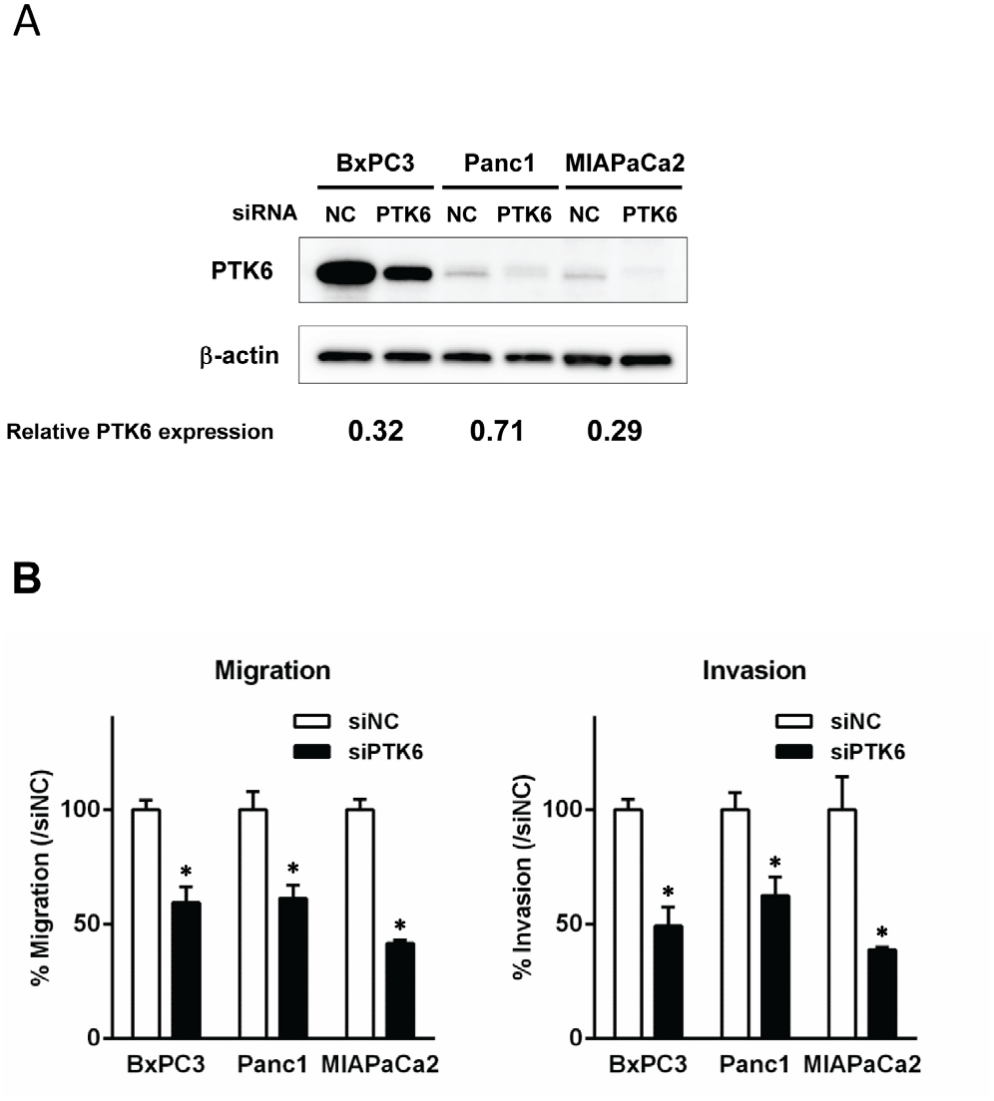
PTK6 gene silencing reduces cell migration and invasion of pancreatic cancer cells. ***A*,** PTK6 gene silencing. Cells were transfected with PTK6-specific siRNA (siRNA-PTK6) or negative control-siRNA (siRNA-NC) for 96 hours. Knockdown of PTK6 expression was comfirmed by Western-blotting. The numbers at the bottom indicate the relative intensity of the bands for PTK6 to corresponding controls (normalized by β-actin). ***B*** **,** ***C*** **,** The effect of PTK6 gene silencing on cell motility (B) and invasion (C). Cells were treated with PTK6-specific siRNA or negative control-siRNA for 48 hours, then subjected to migration or invasion assay using Boyden chambers without or with Matrigel. Gene silencing of PTK6 reduced cellular migration in all 3 pancreatic cancer cell lines (0.59 fold decrease in BXPC3, 0.61 in Panc1, 0.42 in MIAPaCa2, respectively, *p<0.05 by t-test). Similarly, gene silencing of PTK6 reduced cellular invasion in all 3 pancreatic cancer cell lines (0.49-fold decrease in BXPC3, 0.62-fold in Panc1, 0.39-fold in MIAPaCa2, respectively, *p<0.05 by t-test). Each assay was performed in triplicate.

Several studies have suggested that PTK6 influences cellular machinery mediating migration and invasion. [13]–[15] We therefore hypothesized that PTK6 is a key regulator of pancreatic cancer migration and invasion and we evaluated the effect of decreased PTK6 expression on these cell behaviors. After PTK6 expression was suppressed by gene silencing using siRNA (from Ambion) in 3 pancreatic cancer cell lines (Figure 2A), the migratory potential of pancreatic cancer cells were assayed using a Boyden chamber. As shown in Figure 2B, gene silencing of PTK6 significantly reduced migration in all 3 pancreatic cancer cell lines (0.59 fold decrease in BXPC3, 0.61 in Panc1, 0.42 in MIAPaCa2, respectively, *p*<0.05 for each). Cellular invasive potential was similarly assayed using Matrigel-coated Boyden chambers, and invasion was significantly reduced by gene silencing of PTK6 in all 3 pancreatic cancer cell lines, as shown in Figure 2C (0.49-fold decrease in BXPC3, 0.62 in Panc1, 0.39 in MIAPaCa2, respectively, *p*<0.05 for each). These inhibitory effects of PTK6 gene silencing on cell migration and invasion were confirmed by the similar experiments using other siRNA targeting different sequence of PTK6 (from Invitrogen) (Figure S2).

**Figure 3:**
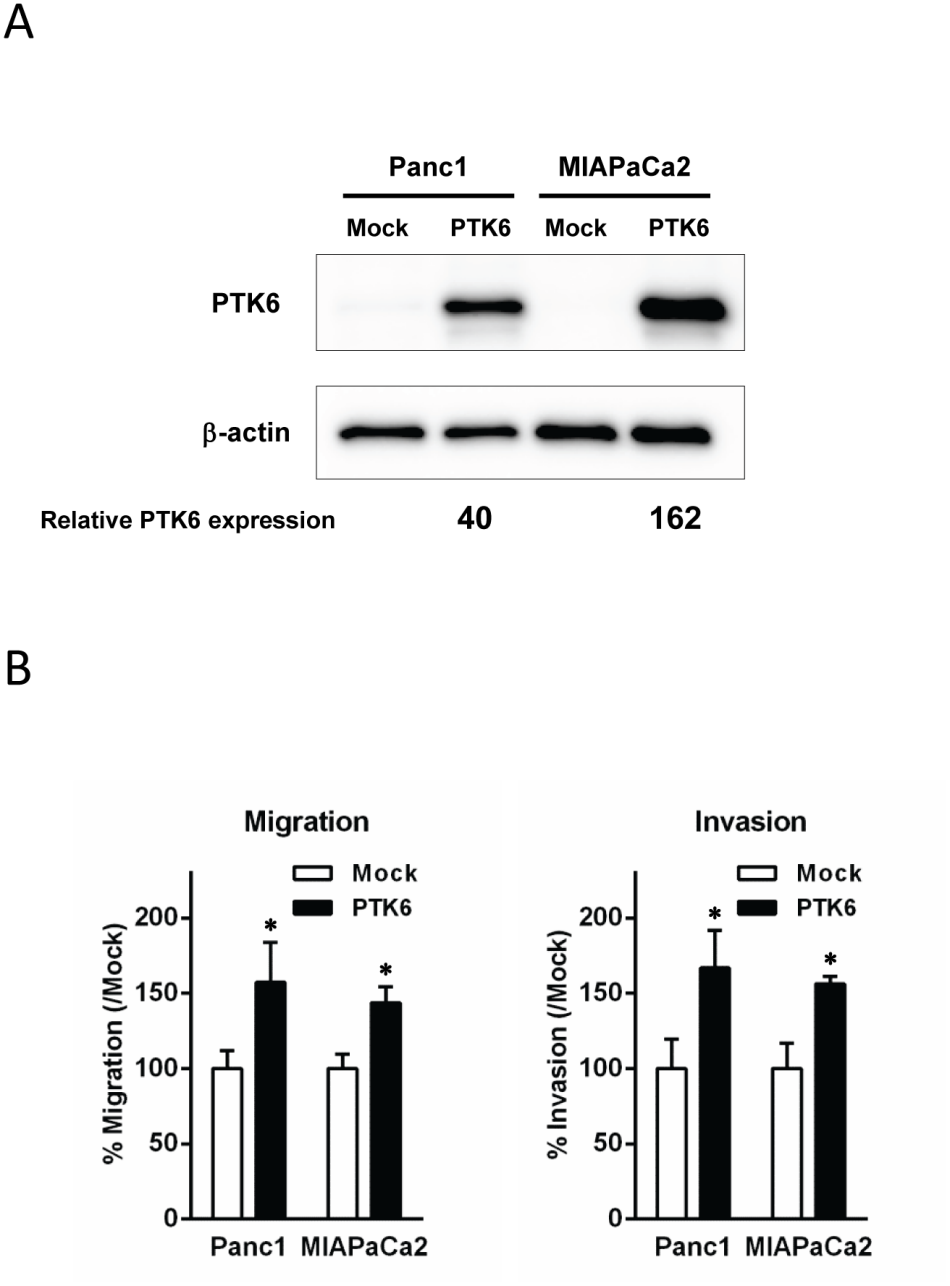
PTK6 overexpression increases cell migration and invasion of pancreatic cancer cells. ***A*,** Overexpression of PTK6. Cells were transfected with pcDNA3.1-Mock (empty vector) or pcDNA3.1-PTK6. Overexpression of PTK6 in stable trasfectant was confirmed by Western blotting in Panc1 and MIAPaCa2 cells, respectively. The numbers at the bottom indicate the relative intensity of the band for PTK6 to the corresponding controls (normalized by β-actin). ***B*** **,** ***C*** **,** The effect of PTK6 overexpression on cellular migration (B) and invasion (C). Established transfectant cells were evaluated with migration and invasion assays. Forced overexpression of PTK6 increased cellular migration in Panc1 and MiaPaCa2 pancreatic cancer cells (1.6-fold increase for Panc1, and 1.4-fold for MIAPaCa2, respectively, *p<0.05 by t-test). Similarly, forced overexpression of PTK6 increased cellular invasion in these 2 cell lines (1.7-fold increase for Panc1, and 1.6-fold for MiaPaCa2, respectively, *p<0.05 by t-test). Each assay was performed in triplicate.

Conversely, we next evaluated the effect of PTK6 overexpression on migration and invasion. An expression vector encoding full length PTK6 cDNA was transfected into Panc1 and MIAPaCa2 cells and stable transfectants overexpressing PTK6 were established for each cell line (Figure 3A). In contrast to the effect of PTK6 gene silencing, PTK6 overexpression increased cellular migration and invasion compared to controls. (1.6/1.7-fold increase in Panc1, and 1.4/1.6 in MIAPaCa2, for migratory and invasive potential, respectively, *p*<0.05) (Figure 3B, 3C) These findings suggest that PTK6 expression in pancreatic cancer is associated with its cellular migratory and invasive potential.

### MAPK/ERK Signaling is an Critical Mediator in PTK6-related Cell Motility

**Figure 4:**
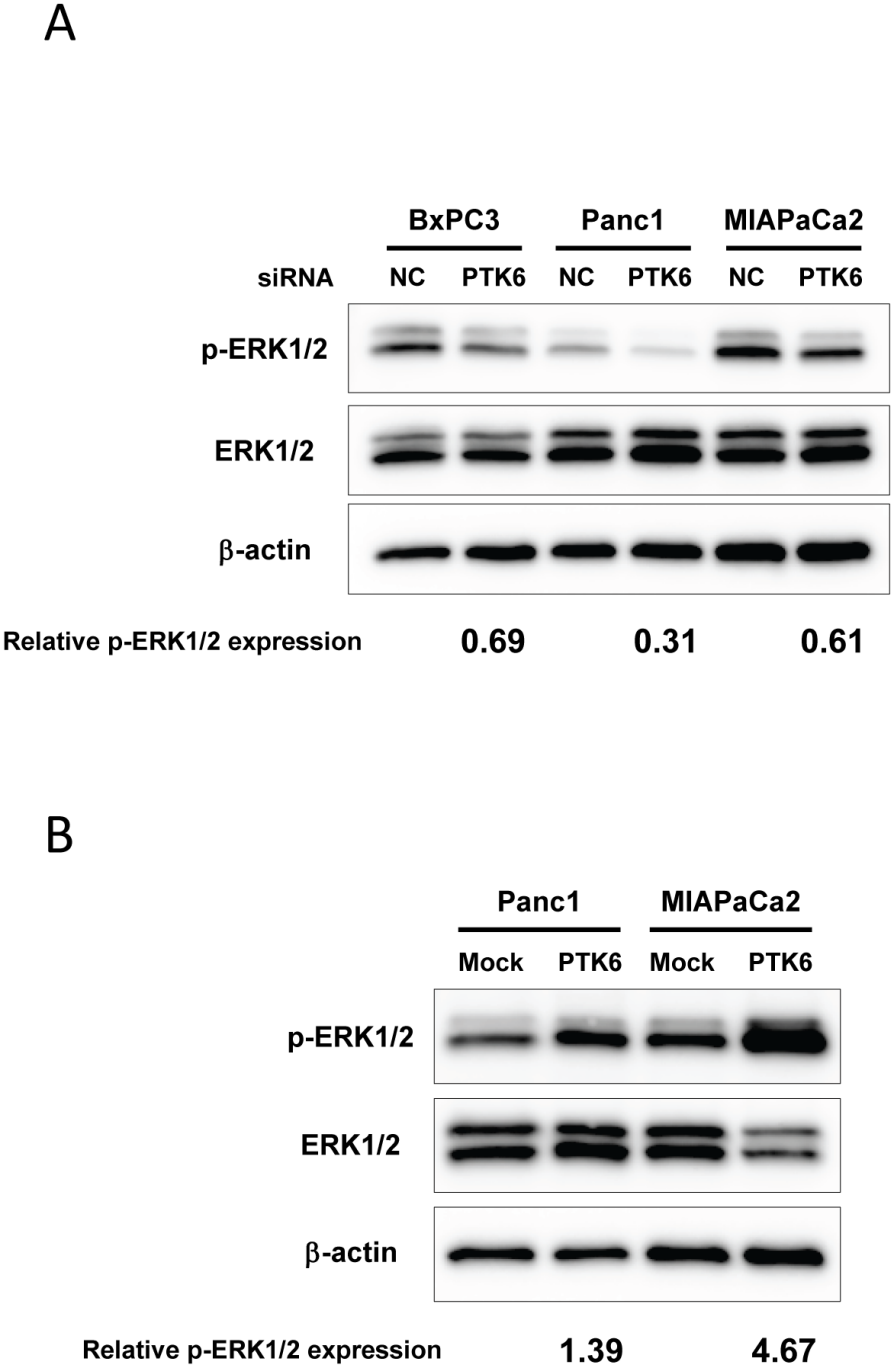
Alteration in PTK6 expression affects ERK1/2 activation. ***A*,** Effect of PTK6 gene silencing on total ERK1/2 and activated (phosphorylated) ERK1/2. Gene silencing of PTK6 reduced the level of phosphorylated ERK1/2 in all 3 pancreatic cancer cell lines. The numbers at the bottom indicate the relative band intensity for pERK1/2 to corresponding controls (normalized by total ERK1/2). ***B*** **,** Effect of PTK6 overexpression on total ERK1/2 and activated ERK1/2. Forced PTK6 overexpression induced activation of ERK1/2 in both Panc1 and MiaPaCa2 cells. The numbers at the bottom indicate the relative band intensity for pERK1/2 to corresponding controls (normalized by total ERK1/2).

**Figure 5:**
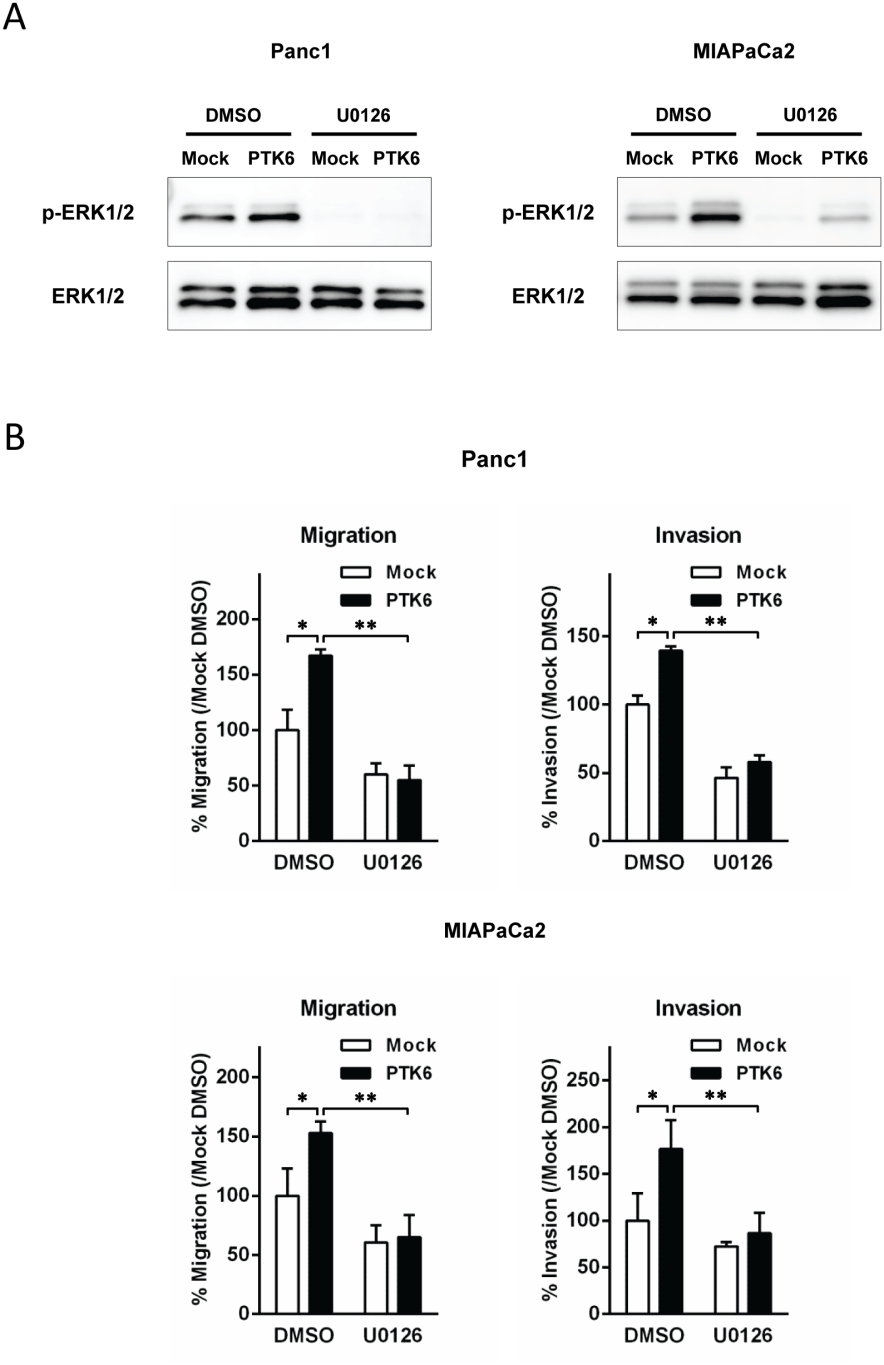
The effect of ERK1/2 inhibition on PTK6-induced cellular migration and invasion. ***A*,** Cells were treated with the specific ERK1/2 inhibitor, U0126 at 10 µM for 24 hours. ERK1/2 phosphorylation was inhibited and PTK6-induced activation of ERK1/2 was no longer detected in Panc1 or MIAPaCa2 cells. ***B*** **,** The effect of ERK1/2 inhibition on cellular migration (*left*) and invasion (*right*) ERK1/2 inhibition not only reduced basal migration and invasion in Panc1 and MIAPaCa2 cells, but abolished the effect of PTK6-overexpression on migration and invasion. *p<0.05 vs corresponding control with DMSO vehicle (by *t*-test). **p<0.05 vs PTK6-overexpressed cells with DMSO vehicle (by *t*-test). Each assay was performed in triplicate.

To elucidate the mechanism in which PTK6 regulates cellular migration and invasion, we explored signaling pathways downstream to PTK6. Several signaling molecules have previously been reported to be downstream of PTK6, including STAT3 [16], Akt [17], [18], Erk5 [15], [19], p38 MAPK [19], and ERK1/2 [20] **(reviewed in Ostrander 2010)**. When PTK6 expression was manipulated using siRNA, the phosphorylation of STAT3, Akt, and p38 MAPK were not altered consistently among 3 cell lines we tested (Figure S1). Activation of ERK5 was not detectable in our studies (data not shown) In contrast, activated (phospholylated) ERK1/2 was significantly reduced in all PTK6-gene-silenced pancreatic cancer cells compared to their corresponding control cells, while forced overexpression of PTK6 induced activation of ERK1/2 in both Panc1 and MIAPaCa2 cells (Figure 4A.B). ERK1/2 therefore emerged as the candidate for the mediator of PTK6-regulated cellular motility in pancreatic cancer and we accordingly sought to determine whether the effects of PTK6 on cellular migration and invasion depend on ERK1/2 activity. We used the selective inhibitor of ERK1/2, U0126 to inhibit ERK1/2 activity. As shown in Figure 5A, PTK6-induced ERK/1/2 activation were completely blocked by U0126 at 10 µM. When we assayed cellular migration and invasion of Panc1 and MIAPaCa 2 cells in the presence of U0126, U0126 reduced basal cellular migration and invasion and abolished the ability of PTK6 over-expression to stimulate them (Figure 5B). This suggests that PTK6 regulates cellular migration and invasion through ERK1/2.

## Discussion

Although there is an accumulating evidence that PTK6 is aberrantly expressed in various epithelial cancers, the implication of PTK6 expression and its role in the biological behavior of cancers are controversial and poorly defined. In fact, the role and function of PTK6 is believed greatly dependent upon the context in which PTK6 is expressed. For example, PTK6 has been extensively studied in breast cancers for its *pro*-oncogenic roles including cell cycle progression [21], angiogenesis [22], anoikis resistance [23] and cellular migration [13], while PTK6 has been shown to work *anti*-oncogenically in esophageal cancer [24]. To our knowledge, this is the first study investigating the expression and the function of PTK6 in pancreatic cancer cells.

Our key finding in this study is that PTK6 overexpression increases cellular migration and invasion and that PTK6 gene silencing, in contrast, decreases them in pancreatic cancer cells. The effects of PTK6 on cellular migration may vary among types of cacners. In the studies of breast cancer cells, PTK6 has been reported to enhance EGF-induced cellular migration [13]–[15], [19], while overexpression of PTK6 was reported to increase cellular invasive potential by inducing epithelial mesenchymal translation (EMT) in prostate cancer cells [25]. In contrast, Ma et al reported that PTK6 decreases the migration of esophageal cancer cells [24]. These apparently opposite reports about the influence of of PTK6 on cancer biology highlight the importance of studies investigating the effects of individual signals in individual types of cancers. It seems likely that the effects of PTK6, like many other signals, depend upon the general kinome in which the PTK6 is expressed. Understanding the kinomic differences between cancers in which PTK6 promotes migration and those in which it inhibits migration may be a fruitful subject for future study. Several downstream effector molecules associated with PTK6 have previously been identified in other cancers. These include p190RhoGAP, Rho, RAS [14], Rac1, Paxillin [13], p38MAPK, and ERK5 [15], [19] in breast cancer, and AKT, GSK3β, and βCatenin [24] in esophageal cancer. In the current study of multiple pancreatic cancer cell lines, most of the molecules that have previously reported to be associated with PTK6 signaling in other cancers did not change in activity by manipulation of PTK6 expression. It is therefore less likely that these molecules play a role in mediating the effect of PTK6 on cellular migration/invasion in pancreatic cancer. Instead, ERK1/2 emerged as a key downstream mediator of PTK6-associated signal which regulates pancreatic cancer invasion. ERK1/2 is a member of the mitogen-activated protein kinase (MAPK) family and is known to influence cellular migration and invasion in various cancers including pancreatic cancer [26], [27]. The mechanisms by which PTK6 overexpression induces ERK1/2 activation has yet to be determined and it is unknown whether PTK6 directly interacts with ERK1/2. Castro and Lange reported that in breast cancer cells the PTK6/ERK5 complex is critical for growth factor induced cell migration, while PTK6 still increases cellular migration by ERK1/2 activation in keratinocytes when ERK5 is knocked down [15]. They speculated that ERK1/2 might serve as an alternative pathway for PTK6/ERK5 signals in some types of cells that lack ERK5. This model would be consistent with our observations since we were not able to detect ERK5 activity in pancreatic cancer cells regardless of PTK6 expression status. However, Xiang et al demonstrated that PTK6 binds directly to Erb B2 and activates ERK1/2 in breast cancer cells. Erb B2 is well known to be overexpressed commonly in pancreatic cancers [28], [29] so this might be a possible mechanism by which forced PTK6 overexpression in pancreatic cancer cells could activates ERK1/2 without ERK5 activity.

It was interesting to note that while PTK6 expression was uniformly undetectable in adjacent histologically normal pancreatic duct epithelial cells, PTK6 levels varied substantially among the pancreatic cancers we studied. Abberant overexpression of PTK6 has been described in other types of cancers and it has previously been suggested that PTK6 may be a useful biomarker to predict outcomes of patients with various cancers including breast, head and neck, ovarian and lung cancers [6], [12], [30]–[33]. The potential prognostic value of variation in PTK6 in pancreatic cancer awaits further study.

In summary, we demonstrated that PTK6 is expressed aberrantly in pancreatic cancer. Furthermore, our results suggest that PTK6 may promote cellular migration and invasion in pancreatic cancer by activating ERK1/2. Further investigation of the PTK6-dependent signaling pathway that drives invasion in pancreatic cancer may highlight the potential prognostic and therapeutic importance of this novel signal in pancreatic cancer.

## Supporting Information

Figure S1 Effect of PTK6 gene silencing on the activity of various molecules in signal pathways; p38, STAT3, and AKT were tested as they were previously reported to be associated with PTK6. The activity of those molecules were not consistently affected by gene silencing of PTK6 in 3 pancreatic cancer cell lines, BXPC3, Panc1 and MIAPaCa2.
(TIF)

Figure S2 ***A*** **,** The effects of PTK6 gene silencing on total ERK1/2 and phosphorylated ERK1/2 using siRNA. Of note, the siRNA used in this experiment has different sequence targeting PTK6 from the siRNA used in the experiment in Figuer 2 and 4. The phosphorylated ERK1/2 were reduced by PTK6 gene silencing in both Panc1 and MIAPaCa2 cells. The numbers at the bottom indicate the relative band intensity for PTK6 and pERK1/2 to corresponding controls, respectively (normalized by β-actin for PTK6 and total ERK1/2 for pERK1/2). ***B*** **,** The effect of PTK6 gene silencing on cell migration (left) and invasion (right). Gene silencing of PTK6 reduced cellular migration in Panc1 and MIAPaCa2 cells (0.65-fold decrease in Panc1, 0.72 in MIAPaCa2, respectively, *p<0.05 by t-test). Furthermore, gene silencing of PTK6 reduced cellular invasion in Panc1 and MIAPaCa2 cells (0.48-fold decrease in Panc1, 0.78-fold in MIAPaCa2, respectively, *p<0.05 by t-test). Each assay was performed in triplicate.
(TIF)
