# Supplementary figures and images for: PTK6 Promotes Cancer Migration and Invasion in Pancreatic Cancer Cells Dependent on ERK Signaling
Source: PLoS One. 2014 May 1;9(5):e96060. doi: 10.1371/journal.pone.0096060 (PMC4006869; doi:10.1371/journal.pone.0096060)

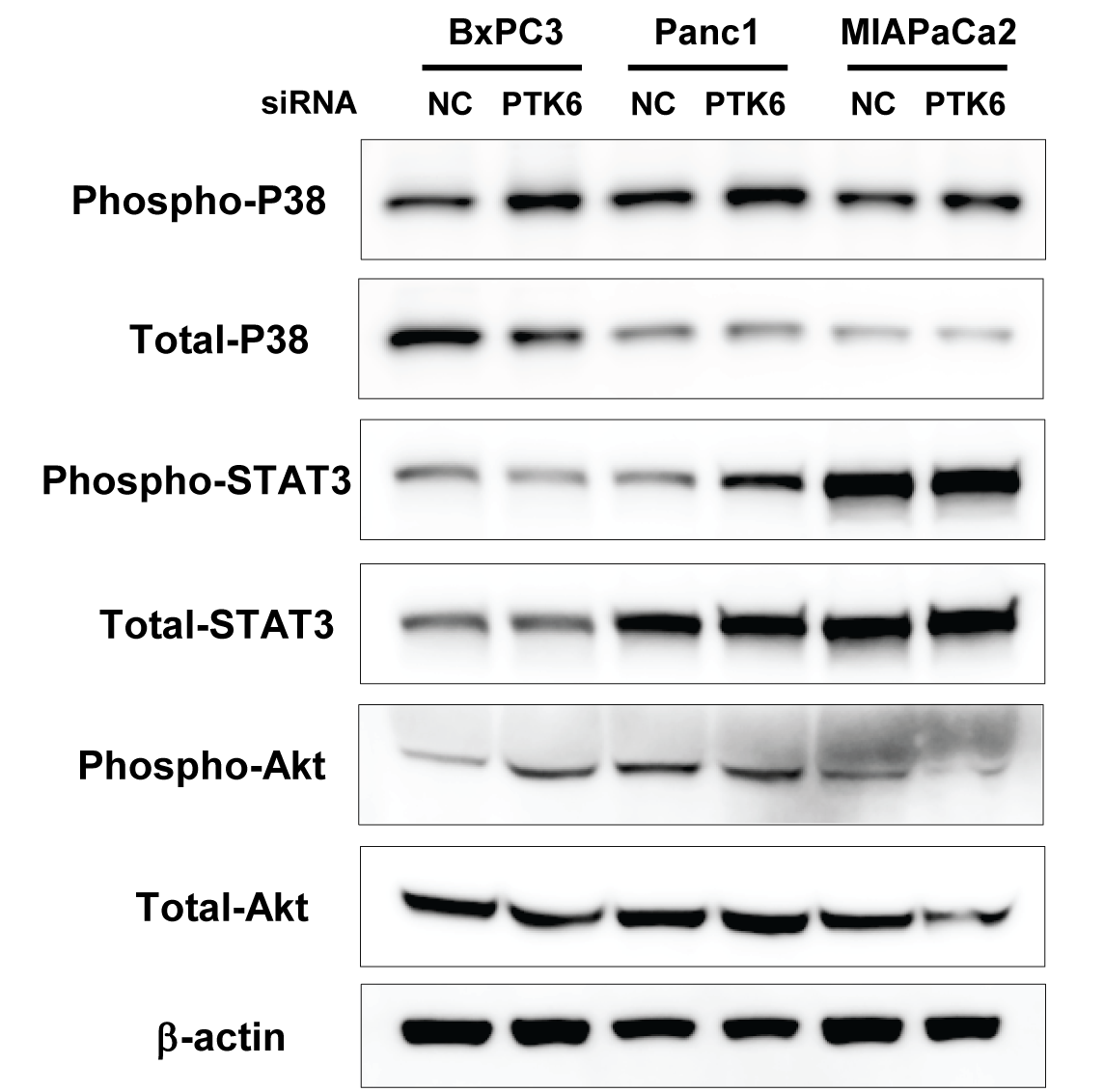

Supplement: Figure S1 — Effect of PTK6 gene silencing on the activity of various molecules in signal pathways; p38, STAT3, and AKT were tested as they were previously reported to be associated with PTK6. The activity of those molecules were not consistently affected by gene silencing of PTK6 in 3 pancreatic cancer cell lines, BXPC3, Panc1 and MIAPaCa2. (TIF) [file pone.0096060.s001.tif]

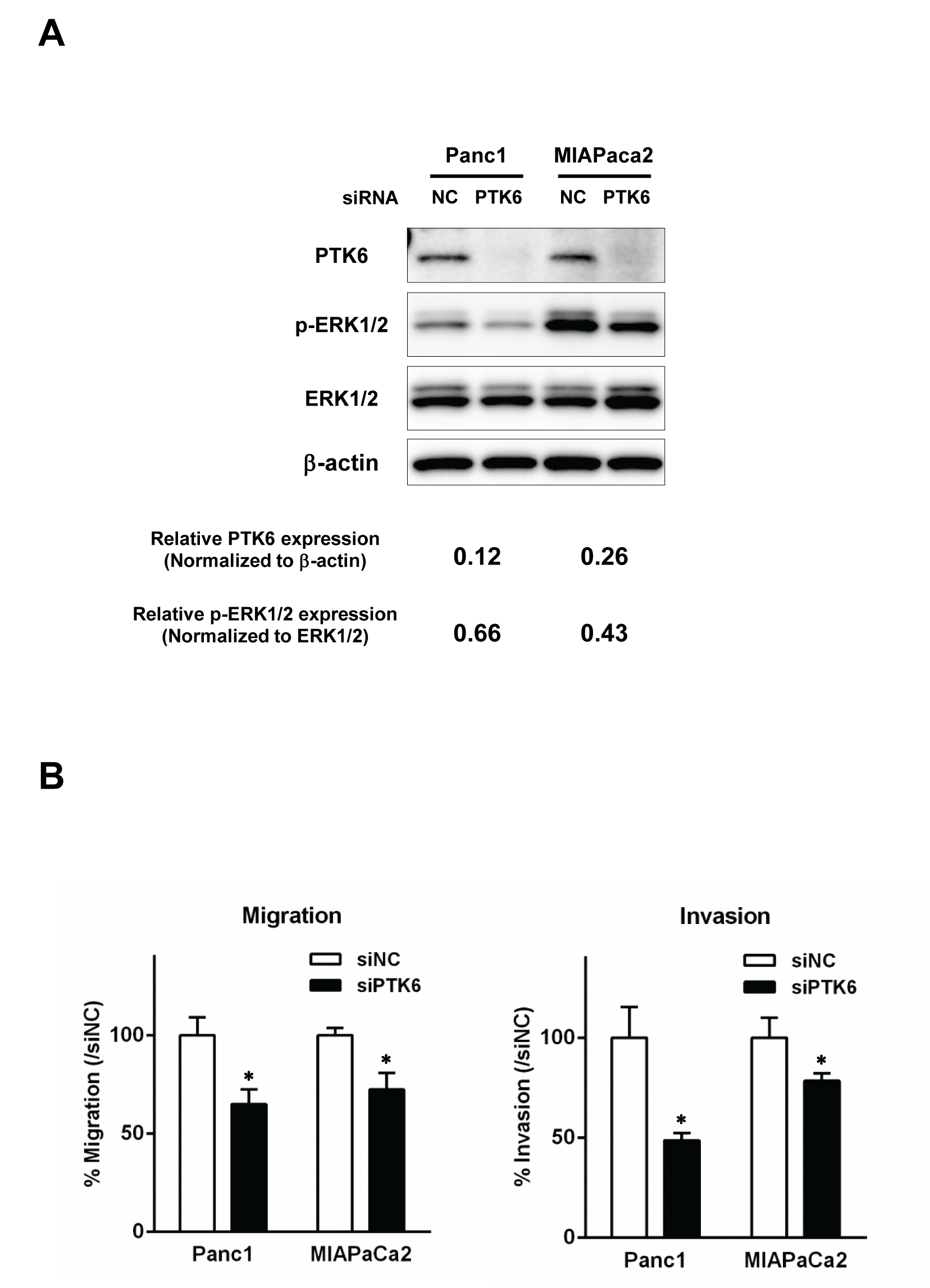

Supplement: Figure S2 — A , The effects of PTK6 gene silencing on total ERK1/2 and phosphorylated ERK1/2 using siRNA. Of note, the siRNA used in this experiment has different sequence targeting PTK6 from the siRNA used in the experiment in Figuer 2 and 4. The phosphorylated ERK1/2 were reduced by PTK6 gene silencing in both Panc1 and MIAPaCa2 cells. The numbers at the bottom indicate the relative band intensity for PTK6 and pERK1/2 to corresponding controls, respectively (normalized by β-actin for PTK6 and total ERK1/2 for pERK1/2). B , The effect of PTK6 gene silencing on cell migration (left) and invasion (right). Gene silencing of PTK6 reduced cellular migration in Panc1 and MIAPaCa2 cells (0.65-fold decrease in Panc1, 0.72 in MIAPaCa2, respectively, *p<0.05 by t-test). Furthermore, gene silencing of PTK6 reduced cellular invasion in Panc1 and MIAPaCa2 cells (0.48-fold decrease in Panc1, 0.78-fold in MIAPaCa2, respectively, *p<0.05 by t-test). Each assay was performed in triplicate. (TIF) [file pone.0096060.s002.tif]
